# Acupuncture for Quality of Life of Patients with Defecation Dysfunction after Sphincter Preserving Surgery for Rectal Cancer: A Systematic Review

**DOI:** 10.1155/2021/7858252

**Published:** 2021-12-15

**Authors:** Guixing Xu, Hanzhou Lei, Yuanfang Zhou, Liuyang Huang, Hao Tian, Zhuo Zhou, Ling Zhao, Fanrong Liang

**Affiliations:** Acupuncture and Tuina School, Chengdu University of Traditional Chinese Medicine, Chengdu, China

## Abstract

**Purpose:**

To evaluate the effectiveness and safety of acupuncture for quality of life of patients with defecation dysfunction (DD) after sphincter preserving surgery for rectal cancer.

**Methods:**

We searched nine online databases from inception to July 1, 2021, and did not restrict the type of language. Then, studies were independently selected by two research team members with screening criteria and risk bias assessment, and the data were extracted. The primary outcome was Quality of Life Questionnaire-Core 29 (QLQ-CR29). The data were then synthesized using the RevMan V.5.2 by random-effects model. Also, we used the standardized mean differences with 95% credible interval (CI) to describe the outcome of the analysis.

**Results:**

A total of 6 randomized controlled trials (RCTs) (with 439 patients) were included in the systematic review, and data from 2 RCTs (with 200 patients) were used in the meta-analysis. Five studies (83%) were judged to have a medium risk of bias, and one was at high risk of bias. For synthesis, data from two medium-risk studies found that acupuncture or electropuncture may improve the QLQ-CR29 with urination (mean difference, −0.39 points; 95%CI, −0.46 to −0.32; *I*^2^ = 34%), abdominal pain (mean difference, −0.71 points; 95%CI, −0.89 to −0.54; *I*^2^ = 9%), stool (mean difference, −0.49 points; 95%CI, −0.77 to −0.20; *I*^2^ = 57%), defecation (mean difference, −0.59 points; 95% CI, −0.85 to −0.33; *I*^2^ = 51%), sexual function (mean difference, 0.93 points; 95% CI, 0.48 to 1.38; *I*^2^ = 90%), and self-feelings (mean difference, 1.04 points; 95% CI, 0.36 to 1.73; *I*^2^ = 94%).

**Conclusion:**

Findings in this study indicate that acupuncture or electropuncture may be effective and safe for DD, but the quality of included studies was very low. So, more large-scale, multicenter, long-term, and high-quality original research is still expected in the future.

## 1. Introduction

Rectal cancer is a common tumor of the digestive system [1] and occurs mostly in middle-aged and elderly people over 50 years of age [2]. Transabdominal anterior resection can retain physiological anus benefit to improve the quality of life and avoid abdominal wall ostomy [3]. However, after sphincter preserving surgery, up to 90% of patients will have a subsequent change in bowel habit known as defecation dysfunction (DD) [4–6], which greatly affects the patients' life quality as there is no effective treatment. Some studies found that acupuncture may be an effective and safe therapy for DD [7–14]. As a form of complementary treatment, acupuncture has been performed on patients with digestive diseases in Eastern nations for a long time but has been limited by the insufficient number of high-quality, well-designed randomized controlled trials. The randomized controlled trials of acupuncture for DD published in past years may change the situation. Therefore, an overall systematic review should be conducted. We performed this systematic review to evaluate the association between acupuncture and DD.

## 2. Methods

We conducted the systematic review in accordance with the Preferred Reporting Items for Systematic Review and Meta-Analysis (PRISMA) statement, and the protocol of this systematic review and meta-analysis has been registered on PROSPERO (https://www.crd.york.ac.uk/PROSPERO, https://clinicaltrials.gov/ct2/show/CRD42019140097), registered on 5 Sep. 2019, and published on BMJ open [15].

### 2.1. Criteria for Inclusion

1. Patients (aged ≥18 years) with DD after sphincter preserving surgery for rectal cancer diagnosed by the Rome III or IV diagnosis criteria for DD.
2. The experimental group is defined as electroacupuncture, floating needle, fine needle, etc. or moxibustion at acupoints or trigger points, Besides, acupuncture plus other interventions will also be included.
3. The control group that will include nonacupuncture techniques, such as, placebo control or other active therapies, is eligible. The acupoint numbers, retaining time and frequency, and treatment sessions will not be limited.
4. We assess the outcome indicators based on some studies concerning the variation in postoperative bowel dysfunction after rectal cancer surgery [16, 17] in this protocol.
5. We included randomized controlled trials (RCTs) randomly dividing the subjects into 2 groups, regardless of whether the blind method was used or not. Multiple-arm trials fitting in the abovementioned criteria are eligible. The data of the first period of crossover trials will also be included.

### 2.2. Primary Outcomes

1. Change in quality-of-life score from baseline to the last available follow-up, measured by the European Organization for Research and Treatment of Cancer Quality of Life Questionnaire-Core 29 (EORTC QLQ-CR29) [16], which was specially used to assess the quality of life of rectal cancer and included six aspects (urination, abdominal pain, stool, defection, sexual function, and self-feeling). Also, a multicenter study collecting symptoms and quality of life in patients with low rectal cancer showed that the higher low anterior resection syndrome (LARS) score was associated with a lower quality of life [16].

### 2.3. Secondary Outcomes

1. Change in LARS scores from baseline to the last available follow-up: the scores of the 5 individual questions are added up to a total score of 0 to 42 points. The LARS score allows the categorization of patients into 3 groups: no LARS (0–20 points), minor LARS (21–29 points), and major LARS (30–42 points). The score has previously been thoroughly validated in a large international study where several psychometric properties of the instrument were evaluated [17, 18].
2. The incidence rate of adverse events: we extract outcomes at all time points measured in the included trials. We pooled available data into short-term (up to two weeks), medium-term (two to six weeks), and long-term (more than six weeks) outcomes, when data are available.

### 2.4. Criteria for Exclusion

1. The experiment group that does not contain the needle and moxibustion will be excluded
2. The study comparing different forms of acupuncture, such as acupuncture versus moxibustion, will be excluded
3. Animal experiment, review, and non-RCTs will be excluded

### 2.5. Search Methods for Identification of Studies

We searched PubMed, the Cochrane Library, EMBASE, Surveillance, Epidemiology, and End Results, the Chinese National Knowledge Infrastructure, the Chinese Biomedical Literature Database, the Wanfang Database, the Chongqing VIP from the inception dates to July 2021. The search strategy of PubMed is presented in appendix I. At the same time, we searched the Chinese clinical registry, the National Institutes of Health Clinical Trials Registry, the Australian New Zealand Clinical Trials Registry, and the International Clinical Trials Registry Platform to find the unpublished literature. Two research team members used the endnote software to manage the search results and independently selected the eligible studies.

### 2.6. Data Extraction and Assessment of the Risk of Bias

We confirmed a standard data extraction form before data extraction. Two reviewers extracted the basic information, trial characteristics, participants, interventions and controls, outcome measurements, results, etc. and conducted the cross check to ensure the accuracy of the results. Meanwhile, the other two reviewers evaluated the risk of bias of included studies according to the Cochrane Collaboration's tool with six aspects (randomly generated sequence number, allocation concealment, blinding of participants and personnel, blinding of outcome assessment, incomplete outcome data, selective reporting, and other bias when required) [19].

### 2.7. Assessment of Heterogeneity and Data Synthesis

We used chi-squared (*X*^2^) in the forest plot to evaluate the heterogeneity of included studies according to the Cochrane Handbook [20] but did not conduct subgroup analysis to explore the reason for heterogeneity on account of not having enough studies. We synthesized all data via RevMan software (V.5.2) with the random-effects model when the heterogeneity *I*^2^ < 75%. We also used the Grading of Recommendations Assessment, Development, and Evaluations (GRADE) system to assess the quality of evidence for each outcome [21].

Due to the low number of studies, subgroup analysis, meta-regression, and sensitivity analysis were not conducted.

## 3. Results

**Figure 1:**
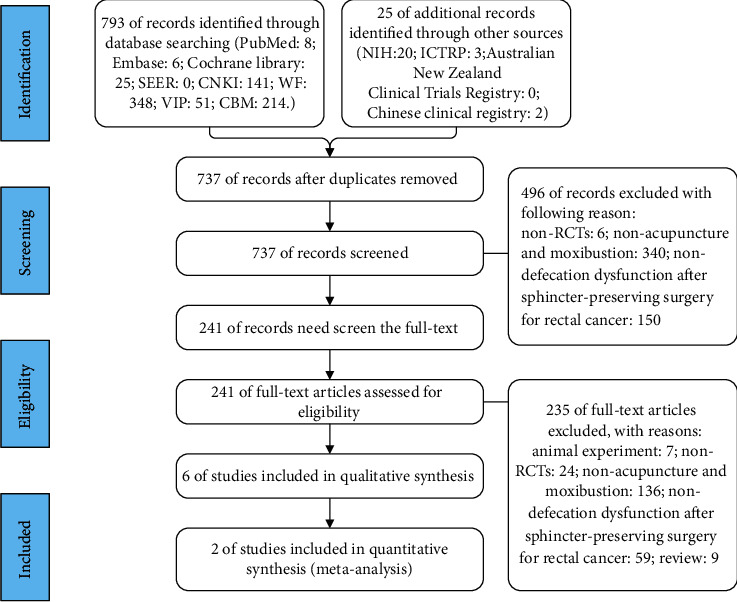
Flowchart of the trial selection process for this systematic review.

A total of 793 articles were identified through database searches, from which 81 duplicate publications (10%) were removed, and 706 articles (89%) were excluded for not meeting the inclusion and exclusion criteria. Six RCTs (1%) were included in the systematic review [10, 11, 22–25] (Figure 1 and Table 1). The study characteristics of these RCTs are summarized in Table 1. Quantitative synthesis was performed with 2 RCTs (33%) by pooling the results through a meta-analysis. All trials involved 439 patients with DD after sphincter preserving surgery for rectal cancer. Three (50%) of the 6 trials had insufficient data [10, 11, 23]. All studies included were conducted in China.

### 3.1. Characteristics of Clinical Studies and Quality of Evidence

Among the 6 RCTs included, all studies were open-label trials. Three (50%) studies involved moxibustion plus other therapies as experiments [10, 23, 24], two (33%) studies used acupuncture plus biofeedback [11, 22], and one study involved electropuncture [25]. Three (50%) studies investigated biofeedback as experiments [10, 11, 22], two (33%) studies were levator ani exercise [24, 25], and one study explored loperamide hydrochloride as treatment [23]. Four (67%) studies compared the acupuncture or moxibustion plus biofeedback with biofeedback or levator ani exercise [10, 11, 22, 24]. The sample size ranged from 23 to 60, and a total of 439 patients were included, with 221 (50.34%) in the experiment group and 218 (49.66%) in the control group. Mean age ranged from 50.92 to 67.82 in the experiment group and 51.43 to 68.18 in the control group. There were 120 (54%) men in the experiment group and 124 (57%) men in the control group. The course of treatment ranged between 14 [23], 18 [11], 30 [10], and 60 [22, 24, 25] days (Table 1).

**Figure 2:**
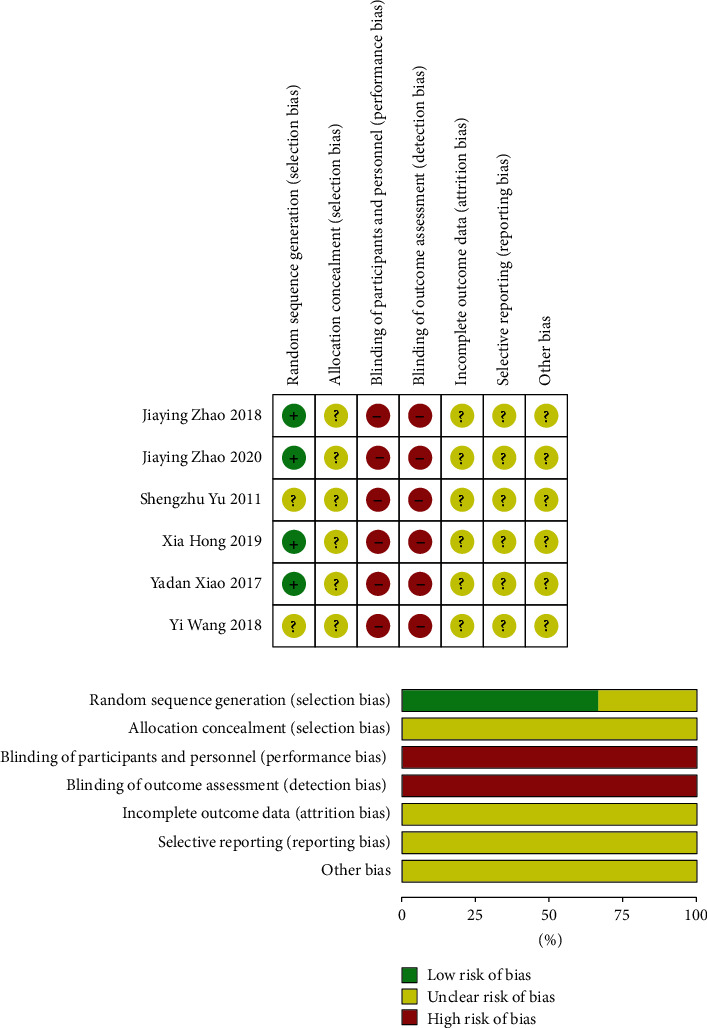
Risk of bias of included studies.

Among the included studies, five [10, 11, 22, 24, 25] (83%) studies had a medium risk bias, and one [23] (17%) had a high risk of bias (Figure 2). All studies involved a high risk of blinding, unclear risk on allocation concealment, incomplete outcome data, selective reporting, and other biases. Four [11, 22, 24, 25] (67%) studies were low risk on random sequence generation, and two [10, 23] (33%) were unclear risk.

### 3.2. Outcome of Acupuncture and Moxibustion

Among the 6 RCTs included, three [10, 11, 23, 24] (50%) studies were not included in the meta-analysis due to data deficiency (the missing data could not be requested by contacting the corresponding author), and only two [22, 25] (33%) studies conducted data synthesis.

**Figure 3:**
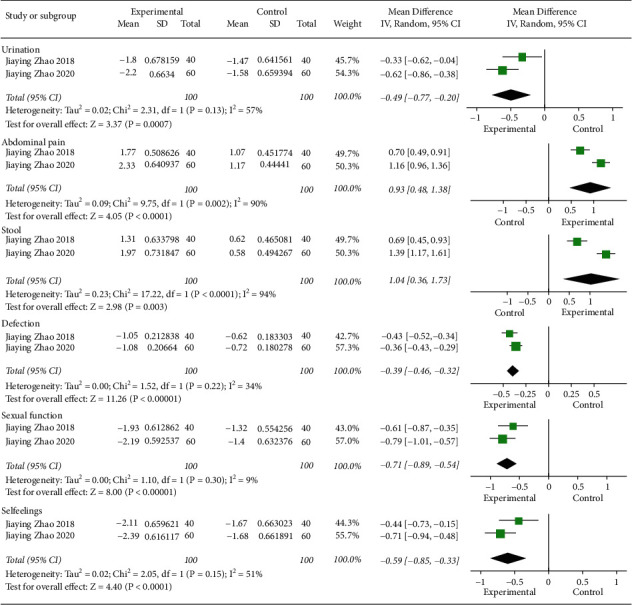
Forest plot of the effect of acupuncture or electroacupuncture on QLQ-CR29.

There were statistically significant pooled benefits of acupuncture plus biofeedback and electropuncture relative to either control, for urination (mean difference, −0.39 points; 95% credible interval (CI), −0.46 to −0.32; *I*^2^ = 34%), abdominal pain (mean difference, −0.71 points; 95% CI, −0.89 to −0.54; *I*^2^ = 9%), stool (mean difference, −0.49 points; 95% CI, −0.77 to −0.20; *I*^2^ = 57%), defecation (mean difference, −0.59 points; 95% CI, −0.85 to −0.33; *I*^2^ = 51%), sexual function (mean difference, 0.93 points; 95% CI, 0.48 to 1.38; *I*^2^ = 90%), and self-feelings (mean difference, 1.04 points; 95% CI, 0.36 to 1.73; *I*^2^ = 94%) (Figure 3).

Although the adverse events of acupuncture were slight, the studies did not report any.

## 4. Discussion

The systematic review included 6 RCTs involving 439 patients with defecation dysfunction after sphincter preserving surgery for rectal cancer, whereas the meta-analysis included 2 RCTs involving 200 patients. Evidence of acupuncture and moxibustion improving defecation dysfunction was found in studies of small sample size and medium quality [22, 25]. Acupuncture may change the patient's bowel habits and help them better control their bowel movements. Research has shown that acupuncture can regulate the intestinal nervous system [26–28], promote the secretion of gastrin and motilin, and improve the blood circulation of the rectum [29] to improve defecation dysfunction. However, high-quality studies were deficient.

This study is the first systematic review of acupuncture treatment of DD after sphincter preserving surgery for rectal cancer. A meta-analysis was conducted with two of the included studies that had enough data, which found that acupuncture or electropuncture may improve the symptoms of DD, with small heterogeneity and moderate risk of bias. The results of the meta-analysis are in keeping with the RCTs. Four studies with different outcomes or insufficient data also indicated acupuncture may be efficient in treating defecation dysfunction. Yi Wang found that adding moxibustion to biofeedback can improve stool score by −1.22 with 95%CI [−1.85, −0.95] compared with biofeedback only [10]. Xia Hong's study also showed that moxibustion in addition to biofeedback can increase the QLQ-C30 9.33 with 95% CI [14.69, 3.97] and improve the LARS score by 4.83 [2.06, 7.60] compared with biofeedback [24]. The study performed by Shengzhu Yu concluded that moxibustion plus Chinese herbs led to better KPS scores compared with drugs [23]. Yadna Xiao found that acupuncture plus biofeedback can improve the stool score by 1.86 with 95%CI [0.88, 2.84] compared with biofeedback [11]. These studies all concluded that acupuncture or moxibustion is beneficial to the treatment of DD after sphincter preserving surgery for rectal cancer.

This study included 6 RCTs, but the data synthesis was conducted with only two of them. The inconsistent outcome indicators were the main reason for exclusion, and the research result reports were not standardized. Many key data are unclear, and the author could not be contacted to provide the original data. Some studies were not registered for clinical experimental research, which led to the lack of methods to obtain research-related information. The following aspects could solve the issues mentioned above: firstly, internationally recognized or the most advanced evaluation indicators should be used as the main outcome indicators. A multicenter study has shown that the LARS score was associated with a lower quality of life [16], so the LARS score may potentially be the primary outcome of defecation dysfunction [24]. Secondly, when reporting research results, all the data collected in the research should be described as completely as possible, and the research results should be written in full accordance with the RCT reporting norm consort [30]. Lastly, all research is encouraged to be preregistered for trials on the corresponding registration website.

In terms of research quality, the included studies were of medium to high risk of bias. No studies reported any allocation concealment and whether to implement blinding, which were the main reasons for high-risk bias. Allocation concealment can mitigate random scores due to various human factors and group measures that cause selection bias [31–33]. Blinding is a measure to avoid implementation bias and measurement bias [34, 35], which helps obtain the most accurate research results. Meanwhile, it remains unclear whether the included studies have selective reporting results and whether there are other biases; these conditions will affect the research results and reduce their value and significance. Therefore, we encourage all clinical research from design, implementation, and results reporting to adopt the most advanced international norms and regulations, which provide the greatest value and significance. Adopting these specific standards is also a responsibility to doctors and patients.

Acupuncture is a complex therapy, and placebo effects exist in acupuncture, which may originate from the interactions between the patient, the clinician, and the treatment environment [36, 37]. All of the included studies did not use sham acupuncture as the control group, so we cannot exclude the placebo effects of acupuncture for DD. Hence, we hope that future clinical studies of acupuncture for DD will include sham acupuncture as the control group.

This study has several limitations, especially the lack of high-quality research, making it impossible to implement the planned published protocol. Secondly, the low quality of the included studies also limits the clinical use of this study's results.

## 5. Conclusions

The findings of this systematic review and meta-analysis suggest that acupuncture or electropuncture may be efficient for DD after sphincter preserving surgery for rectal cancer, but still large-sample, multicenter, long-term, and high-quality RCTs are needed to confirm.

## Figures and Tables

**Table 1:** Characteristics of clinical studies.

Study ID	Location	Study design	Diagnostic criteria	Sample size		Age		Sex (male)		Intervening		Course of treatment		Outcome	Risk of bias
				Experimental	Control	Experimental	Control	Experimental	Control	Experimental	Control	Experimental	Control		
Yi Wang, 2018	China	RCT	(1)	35	35	60.06 ± 8.97	60.07 ± 9.22	18	18	Moxibustion plus biofeedback	Biofeedback	30D	30D	Defecation score	High risk
Xia Hong, 2019	China	RCT	(1)	40	40	50.92 ± 8.77	51.43 ± 9.51	22	24	Moxibustion plus biofeedback	Levator ani exercise	60D	60D	LARS, QLQ-C30	Medium risk
Shengzhu Yu, 2011	China	RCT	(1)	23	23	51.3	53.8	15	14	Moxibustion plus Chinese herb	Drug	14D	14D	KPS	High risk
Yadan Xiao, 2017	China	RCT	(1)	23	20	57.86 ± 10.80	60.00 ± 9.19	12	15	Acupuncture plus biofeedback	Biofeedback	18D	18D	Defecation score	Medium risk
Jiaying Zhao, 2020	China	RCT	(1)	40	40	67.82 ± 9.04	68.18 ± 9.53	17	21	Acupuncture plus biofeedback	Biofeedback	60D	60D	QLQ-C29	Medium risk
Jiaying Zhao, 2018	China	RCT	(1)	60	60	66.73 ± 9.35	65.86 ± 8.74	36	32	Electropuncture	Levator ani exercise	60D	60D	QLQ-C29, CCF-FIS	Medium risk

*Note.* (1) Sphincter preserving surgery for rectal cancer; KPS: Karnofsky performance status.

## Data Availability

No data were used to support this study.

## Supplementary Materials

Supplementary Materials Appendix I presents the search strategy used in the Pubmed database.
