# Supplementary material for: Acupuncture for Quality of Life of Patients with Defecation Dysfunction after Sphincter Preserving Surgery for Rectal Cancer: A Systematic Review
Source: Evid Based Complement Alternat Med. 2021 Dec 15;2021:7858252. doi: 10.1155/2021/7858252 (PMC8694980; doi:10.1155/2021/7858252)
Supplement: Supplementary Materials — Appendix I presents the search strategy used in the Pubmed database. [file 7858252.f1.docx]

| **AppendixⅠ Search strategy used in Pubmed database** | |
| --- | --- |
| **Number** | **Search terms** |
| #1 | randomized controlled trial [All Fields] |
| #2 | controlled clinical trial [All Fields] |
| #3 | randomized [All Fields] |
| #4 | randomised [All Fields] |
| #5 | placebo [All Fields] |
| #6 | randomly [All Fields] |
| #7 | trial [All Fields] |
| #8 | groups [All Fields] |
| #9 | or/#1-#8 |
| #10 | rectal OR rectum [All Fields] |
| #11 | cancer OR carcinoma OR neoplasms [All Fields] |
| #12 | #10 AND #11 |
| #13 | constipation [All Fields] |
| #14 | dyschezia OR obstipation OR constipation OR constipated OR astriction OR costive OR costiveness OR defecation OR defecatory OR defecate OR belly-bound OR oppilated OR oppilate OR oppilation OR Cacation OR ‘bowel movement’ OR ‘hard stool’ OR ‘lumpy stool’ OR constipat* OR ‘impacted stool’ OR ‘rock-like stool’ OR Impaction OR obstipation OR evacuation [All Fields] |
| #15 | delayed bowel movement [All Fields] |
| #16 | bowel AND (function* OR habit* OR movement* OR symptom* OR motility OR stool* [All Fields] |
| #17 | colon transit [All Fields] |
| #18 | intestin* AND (motility OR mobility OR peristalsis OR propulsion OR movement OR emptying [All Fields] |
| #19 | diarrhea OR diarrhoea OR diarrh* [All Fields] |
| #20 | or/#13-#19 |
| #21 | #12 AND #20 |
| #22 | acupuncture [All Fields] |
| #23 | acupuncture therapy [All Fields] |
| #24 | electroacupuncture [All Fields] |
| #25 | electroacupuncture therapy [All Fields] |
| #26 | manual acupuncture [All Fields] |
| #27 | dry needle [All Fields] |
| #28 | moxibustion [All Fields] |
| #29 | acupoint [All Fields] |
| #30 | or/#22-#29 |
| #31 | #9 AND #21 AND #30 |
